# Transient upregulation of EGR1 signaling enhances kidney repair by activating SOX9^+^ renal tubular cells

**DOI:** 10.7150/thno.73426

**Published:** 2022-07-11

**Authors:** Jian-Wen Chen, Meng-Jie Huang, Xiao-Niao Chen, Ling-Ling Wu, Qing-Gang Li, Quan Hong, Jie Wu, Fei Li, Liang-Mei Chen, Yu Dong, Guang-Yan Cai, Xue-Yuan Bai, Zongjin Li, Xiang-Mei Chen

**Affiliations:** 1 Department of Nephrology, First Medical Center of Chinese PLA General Hospital, Nephrology Institute of the Chinese People's Liberation Army, State Key Laboratory of Kidney Diseases, National Clinical Research Center for Kidney Diseases, Beijing Key Laboratory of Kidney Disease Research, Beijing 100853, China.; 2 Beijing Tongren Eye Center, Beijing Tongren Hospital, Capital Medical University, Beijing 100730, China.; 3 Department of Ophthalmology, Chinese PLA General Hospital, Beijing 100853, China.; 4 Nankai University School of Medicine, 94 Weijin Road, Tianjin, 300071, China.

**Keywords:** Acute kidney injury (AKI), Early growth response 1 (EGR1), SOX9, Tubular epithelial cells (TECs), Regeneration

## Abstract

**Background:** Acute kidney injury (AKI) is associated with damage to the nephrons and tubular epithelial cells (TECs), which can lead to chronic kidney disease and end-stage renal disease. Identifying new biomarkers before kidney dysfunction will offer crucial insight into preventive and therapeutic options for the treatment of AKI. Early growth response 1 (EGR1) has been found to be a pioneer transcription factor that can sequentially turn on/off key downstream genes to regulate whole-body regeneration processes in the leopard worm. Whether EGR1 modulates renal regeneration processes in AKI remains to be elucidated.

**Methods:** AKI models of ischemia-reperfusion injury (IRI) and folic acid (FA) were developed to investigate the roles of EGR1 in kidney injury and regeneration. To further determine the function of EGR1, *Egr1^-/-^* mice were applied. Furthermore, RNA sequencing of renal TECs, Chromatin Immunoprecipitation (ChIP) assay, and Dual-luciferase reporter assay were carried out to investigate whether EGR1 affects the expression of SOX9.

**Results:** EGR1 is highly expressed in the kidney after AKI both in humans and mice through analysis of the Gene Expression Omnibus (GEO) database. Furthermore, we verified that EGR1 rapidly up-regulates in the very early stage of IRI and nephrotoxic models of AKI, and validation studies confirmed the essential roles of EGR1 in renal tubular cell regeneration. Further experiments affirmed that genetic inhibition of *Egr1* aggravates the severity of AKI in mouse models. Furthermore, our results revealed that EGR1 could increase SOX9 expression in renal TECs by directly binding to the promoter of the *Sox9* gene, thus promoting SOX9^+^ cell proliferation by activating the Wnt/β-catenin pathway.

**Conclusions:** Together, our results demonstrated that rapid and transient induction of EGR1 plays a renoprotective role in AKI, which highlights the prospects of using EGR1 as a potential therapeutic target for the treatment of AKI.

## Introduction

Acute kidney injury (AKI), a common and severe disease, is associated with a high risk of developing chronic kidney disease (CKD) and end-stage renal disease (ESRD), which is a global health burden with high morbidity and mortality 1, 2. AKI affects all segments of the nephron, tubules, glomerulus, interstitium, and vasculature, but proximal tubular cells are the most commonly injured 3. In response to AKI, the molecular characteristics of tubular epithelial cells (TECs) during this process can drive kidney regeneration or interstitial inflammation and fibrosis 3-5. Identifying new biomarkers before kidney dysfunction might help to detect AKI earlier and will be critical in the development of new therapeutics for AKI treatment 6.

TEC injury is the main pathogenic consequence of AKI 7, and resident TECs have a remarkable ability to proliferate and repair after injury 8, 9. During the repair process, resident TECs extend to repopulate the tubule through a process of dedifferentiation, proliferation, and redifferentiation, restoring the functional integrity of the nephrons 10-12. The regenerative and repair abilities of renal TECs after injury are the key to recovery of renal function in patients with AKI. Sex-determining region Y box 9 (SOX9) is a transcription factor that controls cell fate decisions during homeostasis of a wide range of adult tissues and embryonic development 13. Recently, it has been shown SOX9^+^ cells contribute to renal repair by accelerating the dedifferentiation and proliferation of TECs in the injured kidney 11, 14, 15. However, the upstream and downstream signaling of SOX9 and the mechanisms underlying cellular proliferation and regeneration are still incompletely understood.

Early growth response 1 (EGR1) is an “immediate early” transcription factor that can be rapidly and transiently induced by various cellular stimuli, such as hypoxia, growth factors, and other agents 16, and encodes a protein with a 'zinc finger' motif. EGR1 regulates the expression of many downstream long-term response genes involved in cell differentiation/proliferation, and the inflammatory response 17, 18. EGR was found to bind closed chromatin and rearrange nucleosomes to allow transcription, thus directly activating the expression of genes needed for whole body regeneration in the three-band leopard worm after amputation 19. In normal adult kidneys, the expression of EGR1 was barely detectable 20. By integrated analysis of different high-throughput data and experimental validation, our preliminary research indicated that EGR1 was markedly induced in TECs after injury 20. Previous studies also showed that EGR1 is induced in CKD and functions as a biomarker and pathogenic mediator of kidney fibrosis 21, 22. However, it has not been previously reported whether EGR1 modulates the processes of renal regeneration after AKI.

In this study, our objective was to investigate the functional role of EGR1 in AKI and confirm the transcriptional activation role in the regulation of SOX9. To our knowledge, we demonstrate for the first time that the quick induction of EGR1 in renal tubular cells after AKI is an early response that alleviates injury and thus promotes regeneration and that EGR1 thus plays a renoprotective role in ischemic and nephrotoxic AKI.

## Methods

### Gene Expression Omnibus (GEO) database extraction

The GEO database (https://www.ncbi.nlm.nih.gov/geo/) is a public functional genomics data repository that deposits array- and sequence-based data. To investigate the expression of EGR1 and assess the clinical relevance of EGR1 expression to human and mouse AKI, we used the keyword “AKI, human” or 'AKI, mouse' to search the database and download related gene expression profiles.

### Animals and AKI models

Wild-type C57BL/6 mice (18-22 g) were purchased from the Animal Center of Chinese PLA General Hospital. *Sox9*^fl/fl^ (stock No. 013106), *Sox9*^CreERT2^ (stock No. 018829) and mTmG (also named ROSA mTmG, stock No. 007676) mice were obtained from Jackson Labs. *Slc34a1*^CreERT2^ mice were constructed by Biocytogen Corporation according to methods previously described 23-27. *Egr1^-/-^* mice were bought from GemPharmatech Corporation (Beijing, China). *Slc34a1*^CreERT2/+^ mice were crossed with *Sox9*^fl/fl^ mice to obtain *Slc34a1*^CreERT2/+^:*Sox9*^fl/+^ mice, which were then further crossed with *Sox9*^fl/fl^ mice to obtain *Slc34a1*^CreERT2/+^:*Sox9*^fl/fl^ mice. These mice were housed in a specific pathogen-free facility under a 12-h light/12-h dark cycle with free access to food and water. To induce ischemic AKI, bilateral renal pedicles in the mice were clipped for 30 min using microaneurysm clamps following an established protocol 28. For hydrodynamic-based plasmid delivery 24, 12 h prior to IRI, mice were injected with 20 μg of plasmid DNA in a volume of normal saline (ml) equivalent to 8% of body weight (g) via the tail vein over 5-6 seconds. We constructed a tubule-specific (*Pax8* promoter) *Egr1* overexpression plasmid (GeneCopoeia) to obtain *Egr1* overexpressing (*Egr1^Pax8-OV^*) mice and constructed the* Egr1^Pax8-OV-CFP^* (*pPax8-Egr1-CFP*) plasmid by adding the cyan fluorescence protein (CFP) coding sequence to the *Egr1^Pax8-OV^* plasmid to confirm the efficacy of the hydrodynamic plasmid delivery approach. Mice were sacrificed, blood and kidney tissue samples were collected at the indicated time points after AKI. To induce toxic AKI, mice were subjected to a single intraperitoneal injection of FA at a dose of 250 mg/kg as described elsewhere 29. Mice with CreERT2 were subjected to peritoneal injection of 120 mg/kg tamoxifen once every two days 3 times to activate the catalytic activity of the inducible Cre enzyme. The animal protocol and all animal procedures were reviewed and approved by the Institutional Animal Care and Use Committee of the Chinese PLA General Hospital.

### Quantitative real-time PCR (qRT-PCR)

Total RNA was isolated with TRIzol reagent (Invitrogen, Carlsbad, CA) according to the manufacturer's instructions. The mRNA levels were determined by qRT-PCR analysis on an Applied Biosystems 7500 system (Applied Biosystems, Foster City, CA). The list of primers is presented in **Table S1.**

### Western blot analysis

Kidney tissues or TCMK1 cells were lysed on ice with Radio ImmunoPrecipitation Assay (RIPA) lysis buffer containing 100 μg/mL PhenylMethaneSulfonyl Fluoride (PMSF). The supernatants were collected after centrifugation at 12,000×rpm at 4 °C for 30 min. Approximately 30 μg protein from each sample was added to validate protein expression. Primary and second antibodies are listed in **Table S2.**

### Histology analysis

Samples were isolated at indicated time point. For Hematoxylin-Eosin (H&E) and periodic acid-schiff (PAS) staining, tissues were fixed with 4% formaldehyde, dehydrated and embedded in paraffin. Tissue sections (5 μm) were stained with PAS. Histological examinations of tubular injury were evaluated by acute tubular necrosis (ATN) scores in a blinded manner regarding the grading of tubular necrosis, cast formation, tubular dilation, and loss of the brush border as described previously 20. Fifteen non-overlapping fields (400×) were randomly selected and scored as follows: 0: none; 1: 1 to 10%; 2: 11 to 25%; 3: 26 to 45%; 4: 46 to 75%; and 5: >76%.

For immunofluorescence staining, samples were fixed in 4% paraformaldehyde (PFA) at 4 °C for 18 h, incubated for 2 h in 30% sucrose in phosphate buffered saline (PBS) and embedded in optimal cutting temperature compound (OCT) and then sectioned into 5μm thick slices. The primary and secondary antibodies used are listed in **Table S2.**

### Multiplex immunofluorescence (mIF) staining

MIF staining was conducted by Opal 7-Color Manual IHC Kits (NEL811001KT, Akoya Biosciences, Marlborough, Massachusetts, USA). The slides from formalin-fixed paraffin-embedded kidney tissues were deparaffinized, rehydrated, and subjected to epitope retrieval by boiling in citrate buffer for 20 min at 97 °C. Endogenous peroxidase was then blocked by incubation in 3% H_2_O_2_ for 15 min, and tissue sections were covered with blocking buffer for 10 min at room temperature. Only one antigen was detected in each round, including primary antibody incubation, secondary antibody incubation, and tyramine signal amplification (TSA) visualization, followed by labeling the next antibody after epitope retrieval and protein blocking as before. The antibodies used in this experiment are listed in **Table S2.** The slides were scanned using the PerkinElmer Vectra (Vectra 3.0.5; PerkinElmer, Massachusetts, USA).

### Cell culture and treatment

Primary renal tubular epithelial cells from mice were isolated as previously described 30. Mouse kidney epithelial cell line, TCMK1, was purchased from Beijing Likeli Biotechnology Co. (Beijing, China) and cultured with DMEM/F12 medium (1:1) (Gibco) supplemented with 10% v/v fetal bovine serum (Gibco).

For small interfering RNA (siRNA) or plasmid transfection experiments, we cultured mouse primary renal TECs and kidney epithelial cell line TCMK1 cells to approximately 50% confluence and transfected them using EndoFectin™ Max (GeneCopoeia, China) 12 h before subjecting them to hypoxia/reoxygenation (H/R) injury to mimic IRI. Briefly, cells were incubated in glucose-free medium in hypoxia condition (1.0% O_2_) for 6 h. The cells were then incubated in normal conditions with complete medium for 18 h. In this study, we used a Cytomegalovirus (CMV) promoter plasmid (based on the pReceiver-M02 plasmid, GeneCopoeia) to construct an *Egr1* overexpression plasmid (*Egr1^CMV-OV^*) for use *in vitro* and used the *Pax8* promoter plasmid (*Egr1^ Pax8-OV^*) *in vivo*. A list of siRNA oligosequences used (GenePharma, China) is provided in **Table S3.**

For the *in vitro* scratch wound assay, 12 h after control siRNA or plasmid transfection, TCMK1 cells were subjected to H/R injury. The confluent cell monolayer was scratched using a sterile 200-μl pipette tip, and washed with PBS. Cell images were captured at 0 h and 18 h after scratching with a wide field microscope. Cell migration was calculated as the ratio of the open area after 18 h to the open area at 0 h.

For cell counting kit-8 (CCK-8) assays, a total of 2000 cells per well were seeded in 96-well plates (Corning, NY) and then transfected with siRNAs and plasmids and treated with 25 µmol/L ICG-001 (Wnt/β-catenin inhibitor, SF6827, Beyotime Biotechnology) as appropriate, after which cell (TCMK1 cell) proliferation was measured using the CCK-8 assay (cell counting kit-8, Dojindo, Japan) according to the manufacturer's instructions. Proliferation rates were determined by measuring the absorbance at 450 nm with a microplate reader (Bio-Rad, USA).

For RNA-Seq and analysis, mouse primary renal TECs were placed in 6 cm plates (Corning, NY) and divided into indicated groups. Cells were then collected with 1 mL of TRIzol (Invitrogen, Carlsbad), RNA was extracted and RNA-Seq was performed by Annoroad Corporation (Beijing, China). Gene set enrichment analysis (GSEA) was performed using GSEA 4.0.3 software (http://www.gsea-msigdb.org/gsea/index.jsp). All raw RNA-Seq data were uploaded to the GEO database GSE174812 (SubSeries are GSE174808 and GSE174811).

### Dual-luciferase reporter assay

This experiment can be used to verify the binding of transcription factors (reporter gene) to the promoter of downstream target gene. Briefly, the 2000 bp sequence upstream of the transcription initiation site of *Sox9* containing potential EGR1-binding sites (-AGTGGGGGTGG-) and its mutant sites (-AGTGGGttTGG-) were constructed into an expression vector (*pGL3,* GenePharma, China) containing luciferase to construct the *pGL3-Sox9* or* pGL3-mutSox9* reporter plasmid. The reporter plasmid can regulate the transcriptional expression of Luciferase. The reporter plasmid was then transfected into the 293T cells by using EndoFectin Max (GeneCopoeia, China) according to the manufacturer's instructions, cells were lysed after different treatments (transfected with *Egr1^CMV-OV^* or Control vector). The substrate luciferin was added, and luciferase catalyzed luciferin to emit fluorescence (the strongest wavelength was around 560nm). The fluorescence value can be used to determine the effect of different treatment groups on the transcriptional regulatory element. To avoid errors due to efficiency differences in transfection, Renilla luciferase's reporter plasmid (pRL-CMV) was used as an internal reference (the strongest wavelength was around 465 nm). Luciferase reporter gene expression was measured with a Dual-Luciferase Reporter Assay Kit (Promega, E1910) and a Centro XS LB960 detector (Berthold). Relative firefly luciferase (RFL) activity was obtained by normalizing firefly luciferase activity against Renilla luciferase activity.

### Chromatin Immunoprecipitation (ChIP)

ChIP assay can identify the combination between specific genes and the target protein sequence to reflect the interaction between proteins and DNA. Firstly, the specificity antibody targeting the target protein is used to pull down the DNA fragment which binding to the target protein. Secondly, the protein and the interacted DNA fragment were separate to obtain the DNA fragment, and then qRT-PCR was performed to determine the binding site of the target protein in the genome. In our study, ChIP experiments were performed with a ChIP kit (Cell Signaling Technology, ChIP9003) according to the kit instructions. An antibody against EGR1 for ChIP experiments was purchased from CST (#4154). The qRT-PCR (SYBR Green) was used to measure the amounts of DNA fragments and enrichment efficiency. The primers of the mouse *Sox9* promoter region are 5'-CAGACTCCAGGCGCAGAAG-3' (forward primer) and 5'-GACTTCGCTGGCGTTTACAG-3' (reverse primer).

### Electrophoretic mobility shift assay (EMSA)

EMSA can be used to study the binding of target proteins to specific DNA sequences. The TCMK1 cells were incubated in glucose-free medium in hypoxia condition (1.0% O2) for 6 h, and then incubated in normal conditions with complete medium for 1 h. Nuclear extracts were obtained using a Nuclear and cytoplasmic protein extraction Kit (Beyotime, China) according to the manufacturer's instructions. Single-stranded oligonucleotides were obtained from BGI (Beijing Genomics Institute, China). Double-stranded oligonucleotides were obtained by annealing equal amounts (0.1 mg) of the complementary single-stranded oligonucleotides by heating to 95 °C for 5 min and then gradually cooling to room temperature. Double-stranded oligonucleotides encoding the EGR1 potential binding sequence were 5'-CATCGAAAAGTGGGGGTGGGGGGTTGT-3' and 3'-ACAACCCCCCACCCCCACTTTTCGATG-5'(unlabeled probe), the muted potential binding sequence were 5'-CATCGAAAAGTGGGTTTGGGGGGTTGT-3' and 3'-ACAACCCCCCACCCAAACTTTTCGATG-5' (unlabeled mut-probe). The unlabeled probe was end-labeled with EMSA Probe Biotin Labeling Kit (Beyotime, China). Nuclear extracts were added to 20 µL of binding reactions and incubated for 20 min at room temperature. The EMSA reactions were performed according to the manufacturer's protocol (EMSA/Gel-Shift kit, Beyotime, China). The same unlabeled probe was used as a competitor in the assay. A prominent single super shifted band was observed when nuclear extracts were incubated with an anti-EGR1 antibody.

### Statistical analysis

All data were expressed as Mean ± SEM. Statistical analysis of the data was performed using Graphpad Prism 7.0 (GraphPad, CA, USA). Unpaired Student's t test was used for comparisons between two groups. We used one-way ANOVA corrected with Bonferroni coefficient to compare multiple groups. A* p* value < 0.05 was considered to be significant.

## Results

### Rapid and transient induction of EGR1 both in ischemic and toxic AKI

**Figure 1:**
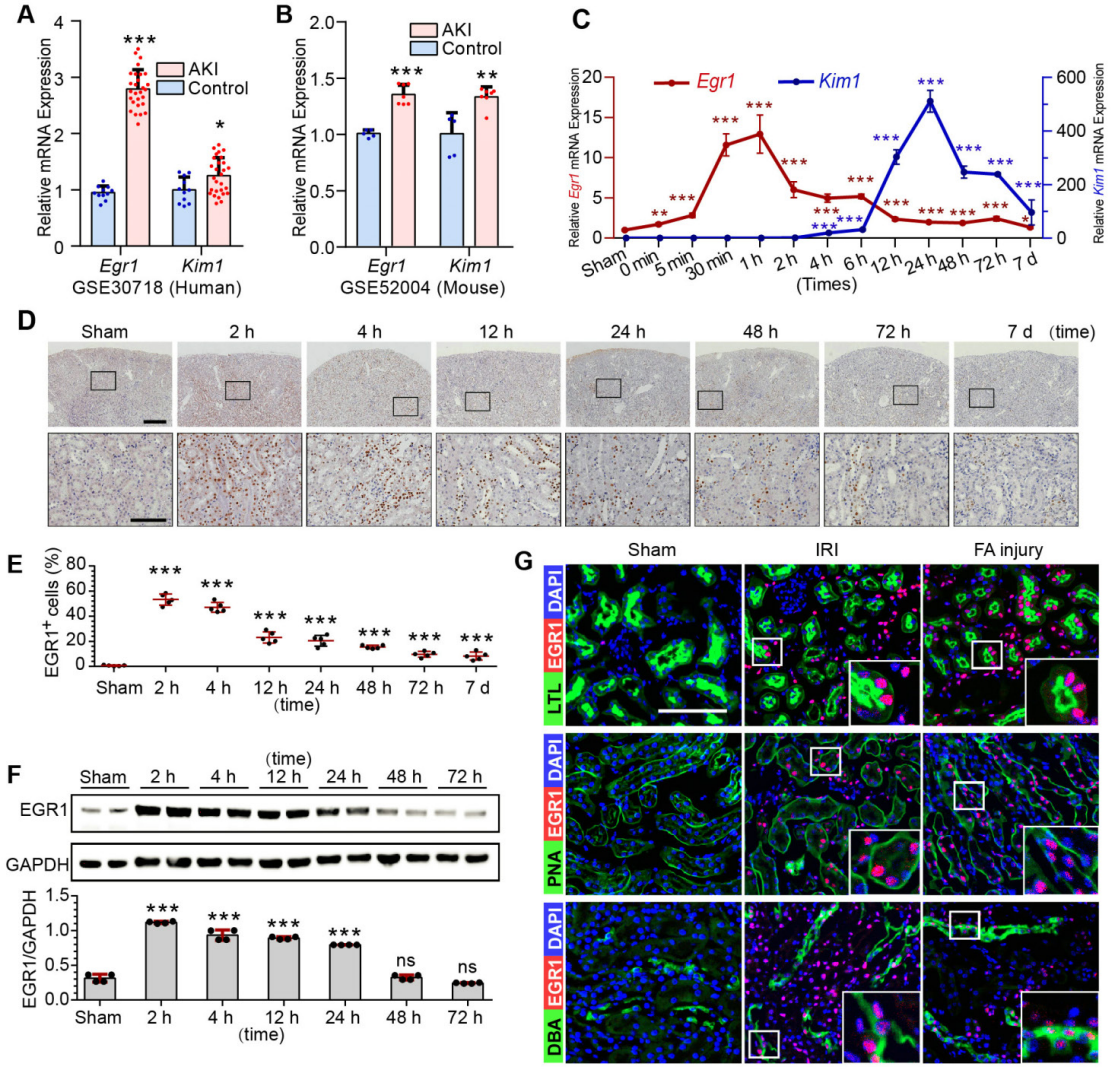
** EGR1 is rapidly and transiently induced in AKI.** (**A**) The mRNA expression of *Egr1* and *Kim1* were significantly upregulated in AKI human sample (dataset GSE30718). (**B**) The mRNA expression of *Egr1* and *Kim1* were significantly upregulated in mouse kidney sample (dataset GSE52004) after IRI. (**C**) The mRNA expression of *Egr1* and *Kim1* in renal tissues at different reperfusion times in our own IRI model. (**D**) Immunohistochemical analysis of EGR1 expression in renal tissues was performed at different time points after I/R injury. Scale bars: 300 µm (upper panel), 100 µm (lower panel). **(E)** Quantification of EGR1 expression. n = 5 mice per group. (**F**) Western blot analysis of renal EGR1 protein expression in injured kidneys at different time points after renal IRI. n = 4 mice per group. (**G**) Co-immunostaining shows the localization of EGR1 after IRI and FA injury. Kidney cryosections 2 h after IRI were double stained with EGR1 (red), and LTL, PNA, DBA (green). Scale bars: 100 µm. ns, no significant; ***p < 0.001. AKI, acute kidney injury; IRI, ischemia-reperfusion injury; qRT-PCR, quantitative real-time PCR; *Kim1*, kidney injury molecule-1; LTL, lotus teragonolobus lectin, a proximal tubule marker; PNA, peanut agglutinin, a henle/distal tubule loop marker; DBA, dolichosbiflorus agglutinin, a collecting duct marker; FA, folic acid. DAPI, 4', 6-diamidino-2-phenylindole.

To investigate EGR1 expression and assess the clinical relevance of EGR1 expression to AKI, keywords “AKI, human” were used to search the Gene Expression Omnibus (GEO) database and downloaded the gene expression profiles GSE30718 31 and GSE145085 32. A significant increase of *Egr1* expression in the AKI human kidney (**Figure 1A**) and human kidney organoid (**Figure S1A**) were observed. Data (GSE164647) from a recently published single cell sequencing study of human kidney organoid 33 revealed that *Egr1* expression was 1.9 times higher in the injured group than that in the control group. Mice mRNA sequencing series GSE52004 34 and GSE98622 35 showed that *Egr1* also increased significantly in mice kidney after IRI (**Figure 1B, S1B**). These results suggest that EGR1 induction is a common feature of AKI both in human and mouse.

Ischemia and toxins are two common causes of AKI in the clinical settings, and we have successfully constructed models of ischemic AKI induced by IRI (**Figure S2A-B**) and toxic AKI induced by folic acid (FA) (**Figure S3**) respectively. Results of qRT-PCR (**Figure 1C**), immunohistochemical staining (**Figure 1D-E**), immunofluorescence staining (**Figure S2B**) and Western blotting (**Figure 1F**) revealed that EGR1 was hardly detected in normal kidney, but was immediately upregulation after IRI. The highest *Egr1* mRNA expression is 30 min to 60 min after IRI, and the highest EGR1 protein expression is 1 h to 6 h after IRI. The expression of EGR1 was earlier than kidney injury molecule-1(KIM1, a marker of kidney injury) in both mRNA and protein level. Similar results were observed in FA-induced AKI (**Figure S3**), suggesting that induction of EGR1 is a common feature that characterizes not only in ischemic AKI but also in toxic AKI. To clarify the tubular location of EGR1, we performed co-staining of the kidney tubules for EGR1 with various markers, lotus teragonolobus lectin (LTL, a proximal tubule marker), peanut agglutinin (PNA, a henle/distal tubule loop marker), dolichos biflorus agglutinin (DBA, a collecting duct marker), aquaporin 2 (AQP2, a collecting duct marker) 36, 37, and Endomucin (EMCN, an endothelial cell marker 38). Immunostaining results indicated that EGR1 could be detected in most renal tubule segments and in some of interstitial endothelial cell of the kidneys with AKI (**Figure 1G & Figure S4**).

### EGR1 decreases tubular injury and drives renal tubule repair and regeneration

**Figure 2:**
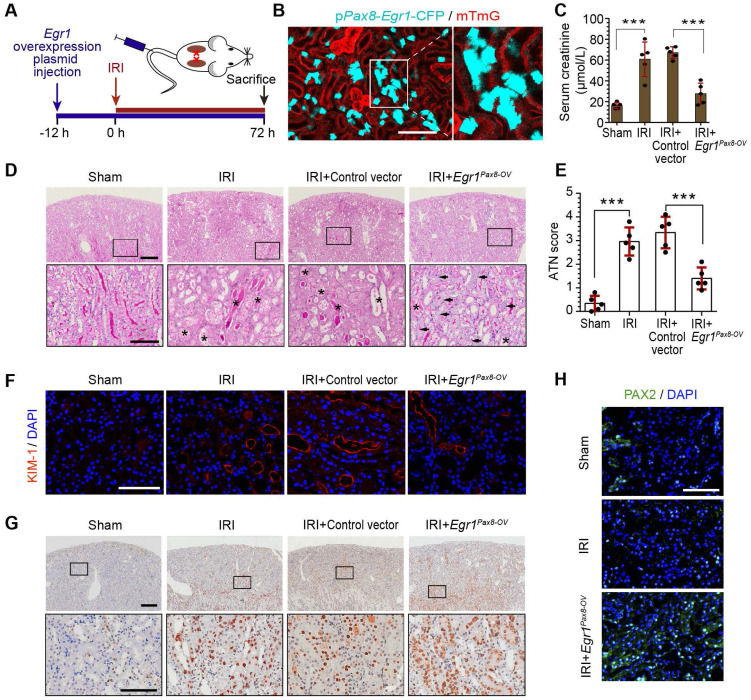
** EGR1 decreases tubular injury and drives renal tubule repair and regeneration after AKI. (A)** Experimental design. The blue arrow indicates mouse tail injection of the *Egr1* overexpression plasmid. The red arrow indicates the establishment of IRI-AKI model. **(B)** CFP expression in renal tubular cells of mTmG mice (tubular cells were all marked red) 24 h after injection of the plasmid *pPax8-Egr1-CFP* (The plasmid transfected cells which were *Pax8* positive will be marked cyan) was traced by two-photon microscopy. This is a direct microscopic observation of living tissue under a two-photon microscope without tissue staining. **(C)** Serum creatinine levels in different groups 3 d after IRI. n = 5 mice per group.** (D)** Representative micrographs of PAS staining show kidney injury in mice injected with Control vector or* pPax8-Egr1* plasmid (*Egr1^Pax8-OV^*) 3 d after IRI. Asterisks in the enlarged boxed areas indicate injured tubules. Arrows in the enlarged boxed areas indicate regenerative cells and cell rearrangement. Scale bars: 300 µm (upper panel), 100 µm (lower panel). **(E)** Quantitative assessment of tubular damage. n = 5 per group.** (F)** Representative immunofluorescence staining of KIM-1 (red) in different groups 3 d after IRI. n = 4 per group. **(G)** Representative micrographs showing PCNA-positive tubular cells in distinct groups after IRI. n = 4 per group. Scale bars: 300 µm (upper panel), 100 µm (lower panel). **(H)** Immunostaining of PAX2 (green) in distinct groups 3 d after IRI. Scale bars: 100 µm. ***p < 0.001. *Egr1^Pax8-OV^*, *Egr1* overexpression plasmid with *Pax8* promoter. IRI, ischemia-reperfusion injury; AKI, acute kidney injury; CFP, cyan fluorescence protein; PAS, periodic acid-schif; SCr, Serum creatinine; ATN, acute tubular necrosis.

To further investigate the function of EGR1 in renal tubule repair after AKI, we constructed *Egr1* overexpressing mice by injecting a *pPax8-Egr1-CFP* plasmid into mice to facilitate kidney tubule-specific *Egr1* overexpression (*Egr1^Pax8-OV-CFP^*). The *pPax8-Egr1-CFP* plasmid contains promoter of paired box 8 (*Pax8*),* Egr1*, and a cyan fluorescence protein (CFP), which can be used to confirm the efficacy of plasmid delivery. **Figure 2A** shows the experimental protocols. By using intravital two-photon microscopy to trace CFP expression in live mTmG mouse, the boost expression of CFP in renal tubules was detected (**Figure 2B**), which indicated that the gene transfection system could effectively increase *Egr1* expression.

To determine the protective function of EGR1 in AKI, mice were subjected to IRI for 12 h after plasmid injection and sacrificed 3 d after surgery (**Figure 2A**). Renal function analysis of serum creatinine (SCr) revealed that EGR1 could significantly ameliorate IRI (**Figure 2C**). PAS staining also showed less morphological injury to the kidneys in mice with EGR1 overexpression (**Figure 2D-E**). Immunostaining of KIM-1 revealed that EGR1 could improve IRI with notable decreasing expression of KIM-1 (**Figure 2F**). Proliferating cell nuclear antigen (PCNA) staining confirmed that EGR1 could increase the proliferation of TECs (**Figure 2G**). PAX2 have been characterized as a marker of dedifferentiated proximal tubule cells 39, 40. Significant upregulation of PAX2 was observed in the cortical area after IRI in *Egr1^Pax8-OV^* mice (**Figure 2H**). Multiplex immunofluorescence (mIF) staining showed that EGR1 can double staining with PAX2 and PCNA in the same cells, while few co-stained with KIM1 3 d after IRI (**Figure S5**). Similar results were observed in analyzing the role of EGR1 in the FA-induced AKI model (**Figure S6**).

### EGR1 deficiency exacerbates kidney injury and inhibits tubule repair

**Figure 3:**
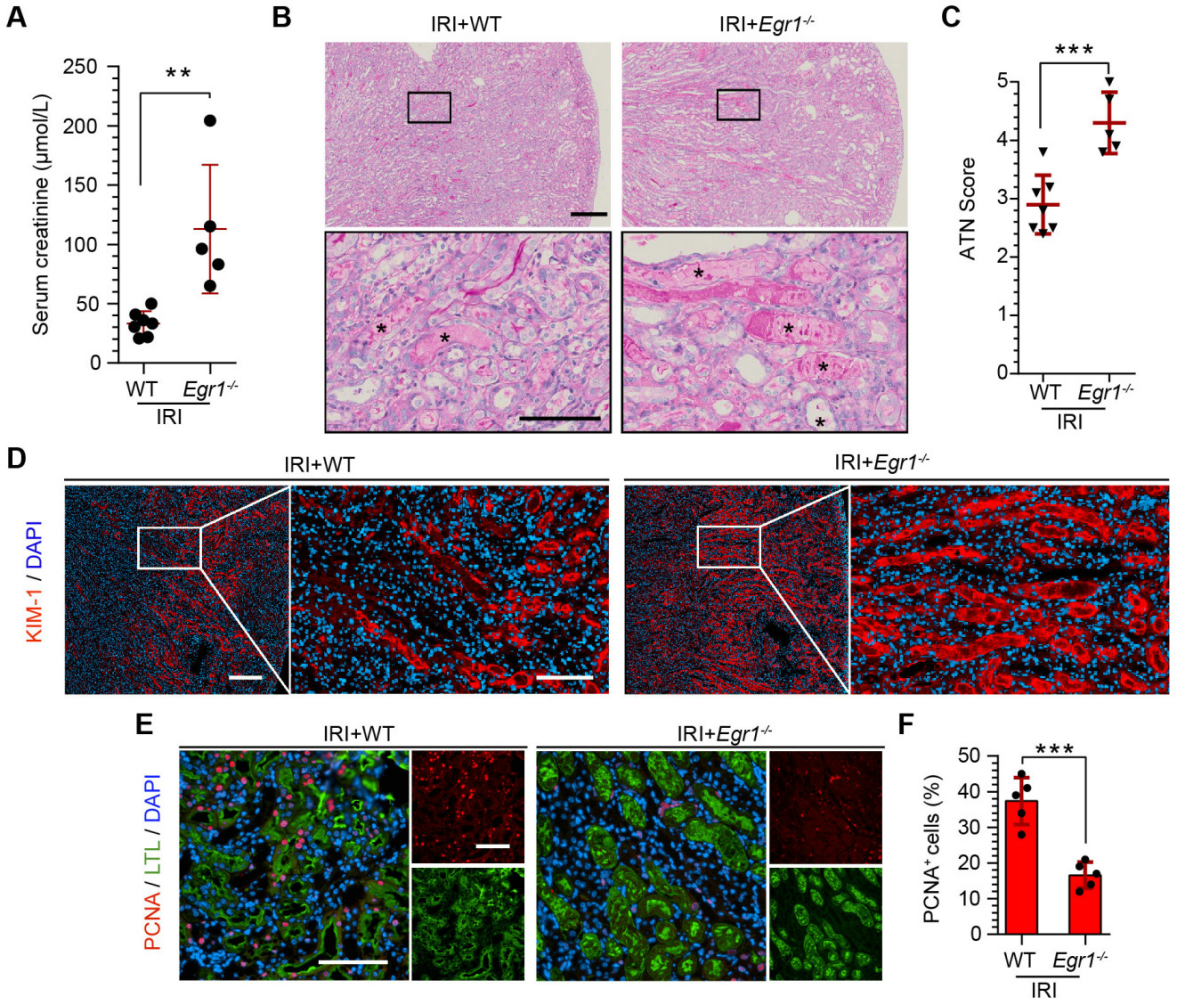
** EGR1 deficiency exacerbates kidney injury and inhibits tubule repair after AKI. (A)** SCr levels in WT and *Egr1^-/-^* mice 3 d after IRI. n = 5-7 per group. **(B)** Representative micrographs after PAS staining show kidney injury in WT and *Egr1^-/-^* mice 3 d after IRI. The asterisks in the enlarged boxed areas indicate injured tubules. Scale bars: 300 µm (upper panel), 100 µm (lower panel). **(C)** The results of a quantitative assessment of morphological damage (ATN score) are presented. n = 5-7 per group. **(D)** Representative immunofluorescence staining of KIM-1 (red) in WT and *Egr1^-/-^* mice 3 d after IRI. Scale bars: 300 µm (left panel), 100 µm (right panel).** (E)** Representative co-immunostaining of PCNA (red) and LTL (green) in different groups 3 d after IRI. Scale bars: 100 µm.** (F)** Quantitative detection of PCNA positive cells in different groups. n = 5 per group. **p < 0.01; ***p < 0.001. SCr, Serum creatinine; IRI, ischemia-reperfusion injury; AKI, acute kidney injury; WT mice, wild-type mice;* Egr1^-/-^* mice,* Egr1* knockout mice; PAS, periodic acid-schiff; ATN, acute tubular necrosis.

To further confirm the protective role of EGR1 in renal tubule repair after AKI, we raised *Egr1* knockout (*Egr1^-/-^*) mice, both *Egr1^-/-^* and WT mice were subjected to IRI and sacrificed 72 h after surgery. The efficacy of *Egr1* knockout was confirmed by immunohistochemistry. *Egr1* was barely detected in *Egr1^-/-^* mice (**Figure S7**). The *Egr1^-/-^* mice were phenotypically normal and showed no appreciable defects in renal morphology or function. However, *Egr1* deficiency significantly aggravated renal IRI with SCr level significantly increased (**Figure 3A**). AKI histopathological damage was evaluated by PAS (periodic acid-Schiff) and KIM-1 staining. PAS staining revealed the cast formation, a marker of renal impairment (**Figure 3B-C**). KIM-1 staining also confirmed the aggravation of kidney injury in *Egr1^-/-^* mice (**Figure 3D**). In addition, similar renal damage was elucidated in cell proliferation as labeled by PCNA (**Figure 3E-F**). The number of PCNA-positive cells was significantly decreased in *Egr1^-/-^* mice compared to WT mice 3 d after IRI. These results confirm the role of EGR1 in kidney regeneration after IR-induced AKI.

### EGR1 promotes the migration and proliferation of TECs

**Figure 4:**
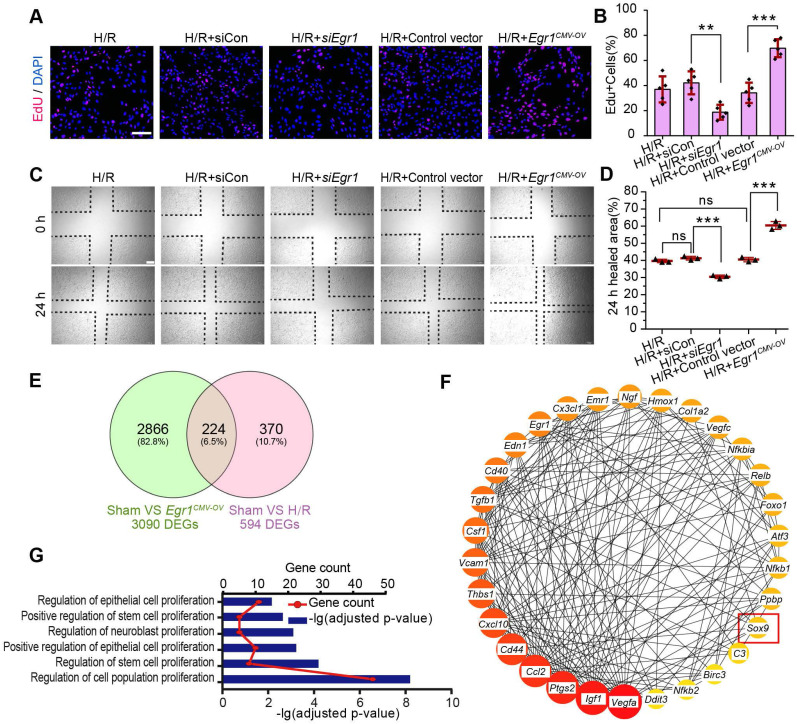
** EGR1 promotes the proliferation and migration of renal TECs after hypoxia/reoxygenation injury *in vitro*. (A)** Representative immunofluorescence staining of EdU (red) in TCMK1 cells after H/R injury. Scale bars: 100 µm. **(B)** Quantitative data showing the number of EdU-positive cells (%) in different groups after H/R injury. n = 5 per group. **(C)** A scratch wound assay was used to detect the migrate ability of TCMK1 cells in *Egr1^CMV-OV^* and si*Egr1* group. Scale bars: 100 µm. **(D)** Quantification of the scratch wound assay data from C., n = 3 per group.** (E)** Mouse primary renal tubular epithelial cells were isolated and treated with H/R (H/R group) or *Egr1* overexpression plasmid (*Egr1^CMV-OV^* group) or not subjected to treatment (Sham group) respectively, each group contained three samples. After sending RNA sequencing (RNA-Seq), a total of 224 overlapping genes were found in the Venn diagram of DEGs in the Sham group vs. the *Egr1^ CMV-OV^* group and the Sham group vs. the H/R group. **(F)** The PPI network of the 30 genes hub genes was visualized with Cytoscape. Among these 30 hub genes, *Sox9* reflects the positive regulation of epithelial cell proliferation (GO:0050679). **(G)** GO analysis of the 224 overlapping genes. ns, no significant; **p < 0.01; ***p < 0.001. H/R, hypoxia/reoxygenation; *Egr1^ CMV-OV^, Egr1* overexpressing plasmid with CMV promotor; siCon, the negative control small interfering RNA; si*Egr1*, small interfering RNA against *Egr1*; DEGs, differentially expressed genes; PPI, protein-protein interaction; RNA-Seq, RNA sequencing.

Next, we investigate the role of EGR1 in the regulation of tubular cell proliferation and migration after H/R injury *in vitro*. We used ethynyl deoxyuridine (EdU) methods for labelling dividing cells. Our results revealed an increased number of EdU-positive cells after hypoxia for 6 h and reoxygenation for 18 h. However, EdU was significantly decreased after silencing Egr1 expression (si*Egr1*), but increased significantly when *Egr1* was overexpressed (**Figure 4A-B**), which suggested that EGR1 promoted the proliferation of renal tubular epithelial cells. Furthermore, the scratch wound assay showed that EGR1 promoted TCMK1 cell migration after H/R injury (**Figure 4C-D**).

### The key molecular networks of EGR1 in kidney repair

To clarify the molecular mechanism by which EGR1 regulates renal TEC proliferation after AKI, we analyzed isolated mouse primary renal tubular epithelial cells by RNA sequencing (RNA-Seq). A total of 3090 differentially expressed genes (DEGs) met the criteria (|log2-fold change (FC) ≥ 0.378 and adjusted false discovery rate (FDR) < 0.05) upon comparison of the Sham group and the *Egr1^CMV-OV^* group. A total of 594 DEGs met the criteria (| log2-FC ≥ 0.263 and FDR < 0.05) upon comparison of the Sham group and the H/R group (**Figure 4E**). By searching the Search Tool for the Retrieval of Interacting Genes/Proteins (STRING) database (http://string-db.org/), 130 of the 224 overlapping DEGs met the criterion of a combined score > 0.4. Hub genes were further extracted using the cyto-Hubba 41 plugin in Cytoscape software 42 as previously described 20. The top 30 genes were identified (**Figure 4F**) and 22 genes were associated with the regulation of cell population proliferation (**Figure 4G**). Among these hub genes, *Sox9* is associated with the positive regulation of epithelial cell proliferation. It has been reported that *Sox9* was required for kidney regeneration 10. In brief, our results collectively indicate that EGR1 may drive *Sox9* pathway activation in kidney repair after AKI.

### EGR1 promotes *Sox9* expression *in vivo* and *in vitro*

**Figure 5:**
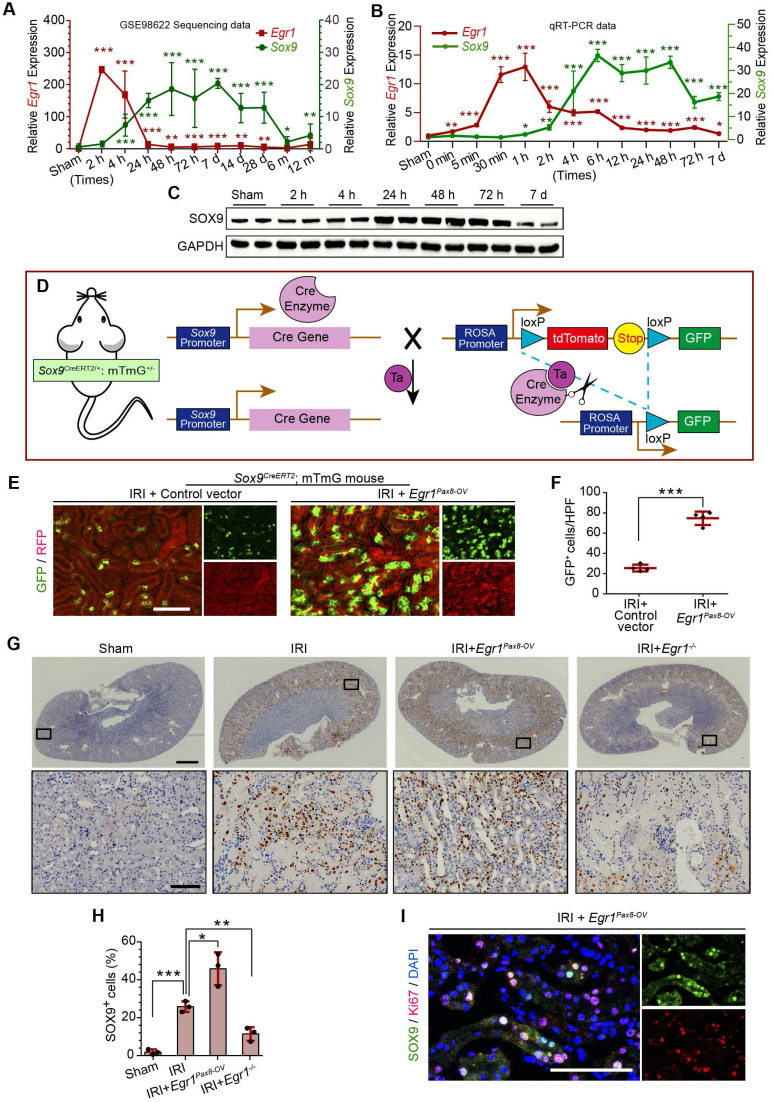
** EGR1 promotes SOX9 expression after AKI *in vivo*. (A)** Expression of *Sox9* and *Egr1* mRNA in mouse kidney tissue at different time points after IRI in the GSE98622 sequencing data.** (B)** The qRT-PCR results of *Sox9* and *Egr1* mRNA expression in mouse kidney tissue at different time points (including the time points within 2 h of reperfusion) after IRI in our own AKI model.** (C)** Western blot analysis shows SOX9 protein levels in injured kidneys at different times after IRI. **(D)** The schematic diagram of how mTmG is regulated by* Sox9^CreERT2^* in *Sox9*^CreERT2^; mTmG^+/-^ mouse. **(E-F)** Representative micrographs **(E)** and number of GFP (green) positive cells **(F)** in *Sox9*^CreERT2^; mTmG^+/-^ mice at 3 d after IRI. n = 4 mice per group. Scale bars: 100 µm. **(G-H)** Immunohistochemical analysis **(G)** and quantitative** (H)** SOX9 expression data in different groups after renal IRI. n = 3 per group. Scale bars: 1 mm (upper panel), 100 µm (lower panel). **(I)** Co-immunostaining of Ki67 (red) and SOX9 (green) in the kidneys of mice injected with the *Egr1^Pax8-OV^* plasmid 3 d after IRI. Scale bars: 100 µm. AKI, acute kidney injury; qRT-PCR, quantitative real-time PCR; *Egr1^Pax8-OV^*, *Egr1* overexpression plasmid with *Pax8* promoter;* Egr1^-/-^*mice.,* Egr1* knockout mice. *p < 0.05; **p < 0.01; ***p < 0.001. IRI, ischemia-reperfusion injury; GFP, green fluorescent protein; RFP, red fluorescent protein; HPF, High power field.

In the GEO dataset of GSE98622 35,* Sox9* mRNA was initially upregulated in renal tissue 4 h after IRI and kept expression for 6 months, whereas *EGR1* expression restored to normal at 24 h after IRI (**Figure 5A**). The similar results were found in our own IRI-AKI model by qRT-PCR analysis (**Figure 5B**). Western blot analysis results revealed that SOX9 was markedly upregulated 24h after IRI (**Figure 5C**), with the highest expression from 24-48 h. The delayed expression of *Sox9* indicates that *Egr1* could be upstream element of the *Sox9* gene. To verify this hypothesis, we delivered a tubule-specific* Egr1* plasmid (*Egr1^Pax8-OV^*, promoted by *Pax8*) into kidneys of *Sox9^CreERT2^:mTmG*^+/-^ mice (**Figure 5D**) 12 h before IRI. As shown in **Figure 5E-F**, the GFP^+^ cells, which represent SOX9*^+^* renal tubular cells, were upregulated significantly by *Egr1* overexpression. Furthermore, immunohistochemical assays revealed that SOX9 expression was up-regulated after IRI and *Egr1* overexpression increased SOX9 expression, but *Egr1* knockout decreased SOX9 expression (**Figure 5E-F**). The majority of SOX9^+^ cells (81.31%) were also Ki67^+^ (**Figure 5G**), indicating that SOX9*^+^* renal cells are the predominant proliferative cell population when *Egr1* overexpression. Similar results were observed in the FA-induced AKI model (**Figure S8**).

### EGR1 mediates SOX9 expression by binding to the promoter of *Sox9*

**Figure 6:**
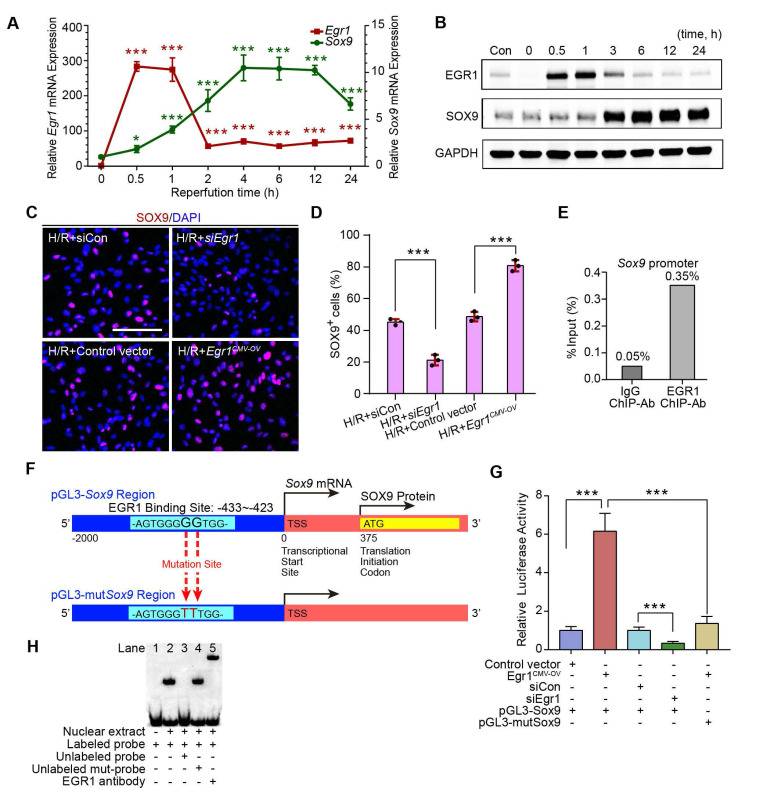
** EGR1 promotes SOX9 expression *in vitro* and mediated SOX9 expression by binding the *Sox9* promoter. (A)** mRNA and **(B)** protein levels of *Sox9* and *Egr1* in TCMK1 cells after reoxygenation for different durations. *p < 0.05 versus the 0h group, ***p < 0.001 versus the 0 h group; ns, no significant. n = 3 per group. **(C-D)** Representative immunofluorescence staining(red) **(C)** and quantitative SOX9 data **(D)** in distinct groups after H/R injury in TCMK1 cells. n = 3 per group. Scale bars: 100 μm. **(E)** ChIP enrichment rate for the *Sox9* gene binding site in EGR1.** (F)** Schematic diagram of possible binding sites in the *Sox9* promoter region for the transcription factor EGR1 and mutation sites.** (G)** Relative luciferase activity in different groups determined by a dual-luciferase reporter assay. n = 5 per group. **(H)** The EMSA assay showed a specific binding of labeled probe to EGR1. Lane 1, biotin-labeled probe only; lane 2, biotin-labeled probe and nuclear extracts; lanes 3, biotin-labeled probe, nuclear extracts plus unlabeled competitor probe; lane 4, biotin-labeled probe, nuclear extracts plus unlabeled mut-probe; lane 5, super-shift EMSA assay with the anti-EGR1 antibody. ***p < 0.001. H/R, hypoxia/reoxygenation; Con, Control; siRNA, small interfering RNA; siCon, negtive control siRNA; *Egr1^ CMV-OV^, Egr1* overexpressing plasmid with CMV promotor; si*Egr1*, siRNA against *Egr1*; pGL3-*Sox9*, pGL3 plasmid containing the *Sox9* promoter region; pGL3-mut*Sox9*, pGL3 plasmid containing the *Sox9* promoter region with mutation sites; ChIP, Chromatin ImmunoPrecipitation; EMSA, Electrophoretic mobility shift assay.

The relationship between EGR1 and SOX9 expression was further verified in the mouse kidney epithelial cell line, TCMK1. The qRT-PCR analysis revealed that *Egr1* expression immediately peaked at 0.5 h after H/R, while *Sox9* expression gradually increased at 0.5 h and reached a plateau at 4 h after H/R (**Figure 6A**), which is consistent with Western blot analysis (**Figure 6B**). The immunofluorescence assay showed the increased expression of SOX9 in *Egr1* overexpressing TCMK1 cells after H/R, but decreased expression in *Egr1* knockdown cells (**Figure 6C-D**). Quantitative RT-PCR analysis showed the same results (**Figure S9A-B**). Immunofluorescence staining revealed that EGR1 was co-stained with SOX9 after *Egr1^CMV-OV-CFP^* plasmid transfected in TCMK1 cells which were subjected to H/R (Figure S9C).

To validate the targeting efficacy of EGR1 on *Sox9*, we constructed a double luciferase reporter system. The promoter region of *Sox9* contains multiple potential binding sites for the transcription factor EGR1, which was predicted through Jaspar database (Jaspar.genereg.net) (**Table S4**). Chromatin immunoprecipitation (ChIP) assay showed that the ChIP enrichment efficiency for EGR1 in the *Sox9* promoter region reached 0.35%, about 7 times higher than that of the negative control IgG group (**Figure 6E**). Among these potential binding sites, only 2 were in sense strand. According to the ranking order, we selected the potential binding site in sense chain with the highest score for the double luciferase assay. The reporter plasmid *pGL3-Sox9* was constructed by cloning the 2000 bp sequence upstream of the transcription initiation site of *Sox9* into the plasmid pGL3 (**Figure 6F**). Our luciferase reporter gene assay elucidated that EGR1 significantly increased luciferase activity by targeting *Sox9* promoter-binding region (**Figure 6G**). The EMSA assay showed a specific binding of labeled probe (this potential binding site) to EGR1 (**Figure 6H**). Taken together, EGR1 regulates *Sox9* transcription expression by binding the promoter of the *Sox9* gene after AKI.

### EGR1 requires SOX9 to drive renal tubule repair and regeneration

**Figure 7:**
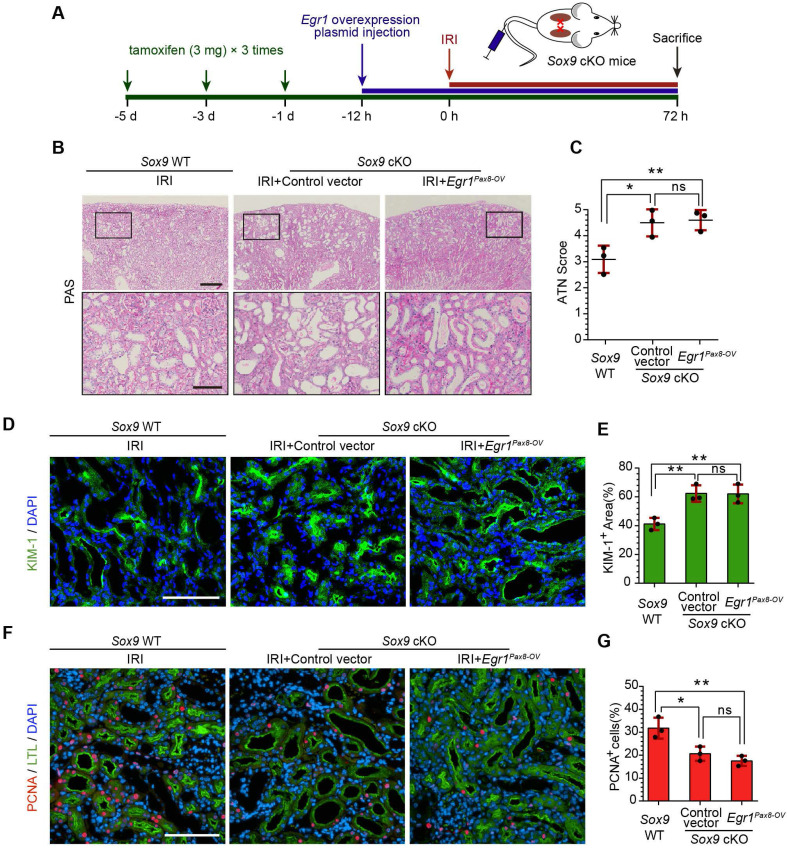
** EGR1 requires SOX9 to drive renal tubule repair and regeneration after AKI. (A)** The experimental design used for the injection of the* Egr1* overexpression plasmid through the mouse tail vein, intraperitoneal injection of tamoxifen, and IRI model construction in*Sox9* WT and *Sox9* cKO mice. **(B)** Representative micrographs of PAS staining show morphological injury in the kidneys of *Sox9* WT or *Sox9* cKO mice injected with Control vector or *Egr1^Pax8-OV^* plasmid 3 d after IRI. Scale bars: 300 µm (left panel), 100 µm (right panel). **(C)** Quantitative assessment of tubular damage. n = 3 per group. **(D-E)** Representative immunofluorescence staining of KIM-1 (green)** (D)** and quantitative detection of the KIM-1 positive area** (E)** in different groups 3 d after IRI. n = 3 per group. Scale bars: 100 µm. **(F)** Representative micrographs show PCNA (red)-positive tubular cells in distinct groups after IRI. Scale bars: 100 µm.** (G)** Quantitative data indicating the number of PCNA-positive cells in *Sox9* WT or *Sox9* cKO mice injected with Control vector or *Egr1^Pax8-OV^* plasmid 3 d after IRI. n = 3 per group. ns, no significant; *p < 0.05, **p < 0.01. IRI, ischemia-reperfusion injury; *Sox9* WT mice,* Slc34a1^CreERT2/+^: Sox9^+/+^* mice; *Sox9* cKO mice*, Slc34a1^CreERT2/+^: Sox9^fl/fl^* mice; PAS, periodic acid-schiff; *Egr1^Pax8-OV^*, *Egr1* overexpression plasmid with *Pax8* promoter.

To further investigate whether EGR1 drives renal tubule repair and regeneration through SOX9, we utilized *Slc34a1*^CreERT2/+^: *Sox9*^fl/fl^ mice in which the *Sox9* gene was specifically knocked out in renal TECs (**Figure S10**). The high efficiency of the Cre recombinant enzyme was shown in **Figure S11A-B**. *Slc34a1*^CreERT2/+^: *Sox9*^+/+^ mice (*Sox9* WT) and *Slc34a1*^CreERT2/+^: *Sox9*^fl/fl^ (*Sox9* cKO) mice were injected with tamoxifen and then injected with Control vector or *Egr1^Pax8-OV^* plasmid 12 h before IRI and sacrificed 3 d after IRI (**Figure 7A**). Compared to *Sox9* WT mice, *Sox9* cKO mice exhibited severe morphological and functional damage to renal tubules, particularly in the cortex and corticomedullary border region.* Egr1^Pax8-^*^OV^ plasmid injection could not rescue this injury (**Figure 7B-C**). Meanwhile, immunofluorescence staining showed an increased KIM-1-positive area in *Sox9* cKO mice at 3 d after IRI, and EGR1 overexpression did not reduce the area of injury induced when *Sox9* was knockout (**Figure 7D-E**). Furthermore, *Sox9* cKO mice showed fewer PCNA positive cells than *Sox9* WT mice, and EGR1 overexpression did not improve the reduced proliferative capacity caused by *Sox9* knockout (**Figure 7F-G**).

### SOX9 promotes the proliferation of TECs via the Wnt/β-catenin pathway

**Figure 8:**
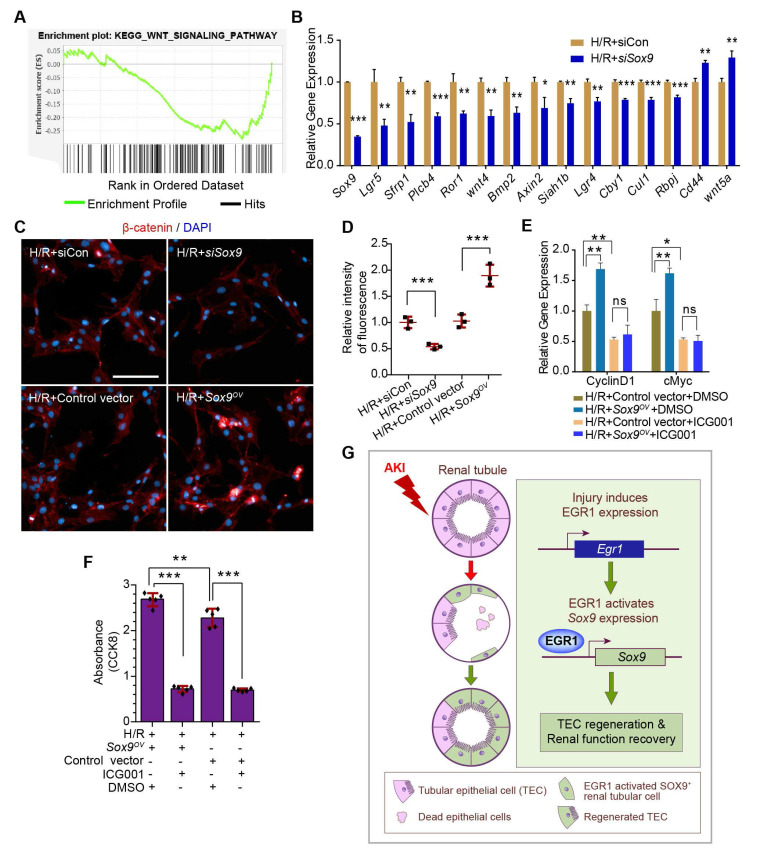
** SOX9 requires the Wnt/β-catenin pathway to drive renal tubule repair and regeneration after AKI. (A)** GSEA of *Sox9*-responsive genes showing enrichment of the WNT signaling pathway in tubular epithelial cells. Normalized enrichment score (NES) = 1.00.** (B)** Relative expression of Wnt/β-catenin pathway-associated genes in si*Sox9* treated mouse primary renal tubular epithelial cells after H/R. **(C-D)** Representative immunofluorescence staining of β-catenin (red) **(C)** and quantitative detection of the β-catenin fluorescence intensity **(D)** in different groups after H/R in TCMK1 cells showing increased expression and nuclear translocalization of β-catenin after *Sox9* overexpression. n = 4 per group. Scale bars: 100 µm. **(E)** Relative expression of Wnt/β-catenin pathway-activated genes (Cyclin D1 and cMyc) in *Sox9*-overexpressing (*Sox9^CMV-OV^*) and ICG001-treated TCMK1 cells after H/R. n = 3 per group. **(F)** CCK-8 assay of TCMK1 cells subjected to different treatments. n = 5 per group. **(G)** The schematic diagram showing the results of the whole study. After ischemic or nephrotoxic AKI, tubular epithelial cells are injured, some cells die, and some cells highly express EGR1. Overexpression of EGR1 increases SOX9 expression by binding the *Sox9* gene promoter, and then SOX9^+^ renal tubular cells regeneration, thereby alleviate tubular injury and promote kidney recovery. ns, no significant; *p < 0.05, **p < 0.01, ***p < 0.001. H/R, hypoxia/reoxygenation; AKI, acute kidney injury; siCon, negtive control siRNA; si*Sox9*, *Sox9*-specific small interfering RNA; *Sox9^OV^*, *Sox9*-overexpression plasmid; ICG001, a Wnt/β-catenin pathway inhibitor; GSEA, Gene set enrichment analysis; CCK-8, Cell Counting Kit-8.

To further investigate the mechanism by which SOX9 promotes proliferation after AKI, *Sox9* expression in primary TECs was knockdown by siRNA and RNA-Seq analysis was applied. Gene set enrichment analysis (GSEA) of SOX9-responsive genes showed significant enrichment of the WNT signaling pathway in primary TECs (**Figure 8A**). The expression of genes associated with the Wnt/β-catenin pathway was significantly altered, and most of these genes decreased significantly after *Sox9* knockdown (**Figure 8B**). A previous study indicated that SOX9 can promote nuclear translocation of β-catenin to activate the Wnt/β-catenin pathway 43. Increased β-catenin expression and nuclear translocalization were observed when *Sox9* was overexpressed, while lower β-catenin expression was observed when *Sox9* was knocked down (**Figure 8C-D**). The Wnt/β-catenin pathway inhibitor ICG001 could reduce the expression of genes activated by the Wnt/β-catenin pathway (Cyclin D1, cMyc) and the proliferation of TCMK1 cells, and *Sox9* overexpression can't rescue the expression of Cyclin D1 and cMyc (**Figure 8E-F**). In brief, SOX9 is required for Wnt/β-catenin pathway to drive renal tubule repair and regeneration after AKI (**Figure 8G**).

## Discussion

In the present study, our results demonstrated that EGR1 is rapidly and transiently in AKI and describe the role of EGR1 in mediating renal epithelial cell regeneration and repair after AKI for first time (**Figure 8G**). Increased expression of EGR1 in renal TECs decreases tubular injury and drives the repair and regeneration of renal tubules both in ischemic and toxic AKI. EGR1 deficiency exacerbates kidney injury and inhibits tubule repair after AKI. The transcription factor EGR1 increases SOX9 expression in renal TECs by directly binding to the promoter of the *Sox9* gene, thus promoting the regeneration of SOX9*^+^* renal tubular cells regeneration by activating the Wnt/β-catenin pathway. We identify the EGR1-SOX9-Wnt/β-catenin axis as a potential target for the prevention and/or attenuation of ischemic and nephrotoxic AKI.

Renal tubules have a remarkable capacity to proliferate and repair after injury 8, 9, and increasing evidences indicate that intrinsic viable epithelial cell migration, proliferation, and dedifferentiation are primarily responsible for tubular regeneration after AKI 44, 45. Applying the IRI or FA injury to *Egr1^Pax8-OV^* and *Egr1^-/-^* mice and the H/R injury to renal cells, we found that EGR1 can improve the regeneration and repair of renal tubules after AKI in mouse model and promote proliferation, dedifferentiation and migration of TECs. Ishibe 46 reported that epithelial dedifferentiation can activate the morphological and transcriptional events involved in cell spreading, migration, and proliferation. The mechanism by which EGR1 promotes dedifferentiation and migration of TECs remains to be elucidated, but could also be related to regeneration and repair.

By performing the RNA-Seq, we found that *Sox9* was one of the 30 *Egr1*-related hub genes and reflects the positive regulation of epithelial cell proliferation. It has been shown that upregulation of *Sox9* is an early cellular response to AKI 10, 11, and *Sox9* has been shown to contribute to renal repair by accelerating the dedifferentiation and proliferation of TECs in the injured kidney 14. SOX9^+^ renal tubular cells play crucial roles in subsequent repair processes after the initial injury phase. Our results showed that *Egr1* and *Sox9* were expressed in sequence by *in vivo* and *in vitro* experiments. A previous report indicated that the canonical Wnt pathway may be actively involved in the kidney-regeneration process 47. Furthermore, SOX9 could promote nuclear translocation of β-catenin to activate the Wnt/β-catenin pathway 43. Therefore, the GSEA of SOX9-responsive genes showed a significant enrichment of the WNT signaling pathway in our RNA-Seq. Our further experiments suggested that SOX9 requires the Wnt/β-catenin pathway to drive renal tubule repair and regeneration after AKI.

Interestingly, our study confirmed that EGR1 expression is an early response by which the kidney alleviates injury and promotes regeneration after AKI, and EGR1 was also reported up-regulated in patients with CKD 21, while EGR1 deficiency attenuated the normal responses of tubular cells to inflammatory factors and alleviated renal fibrosis in tubule interstitial nephritis. Silencing EGR1 could alleviate renal injury in diabetic kidney disease (DKD) 22. Beyond CKD, persistent expression of EGR1 aggravates nephrotic progression, which has also been reported in many other chronic kidney diseases, such as unilateral ureteral obstruction 48 and proteinuric kidney diseases 49. The dual roles of EGR1 may be contradictory. However, many genes, such as SOX9 13, 50-52, epidermal growth factor receptor (EGFR) 53-55, MMP7 30, 56, 57, KIM1 58, 59, and Wnt pathway genes 60-62, also have similar seemingly contradictory effects; that is, they play a role in promoting repair in AKI but also promote fibrosis progression in CKD. This may be because the injury that stimulates AKI is relatively short and mostly transient, while in CKD, sustained injury stimulation and continuous proliferation lead to the occurrence of fibrosis. The above conundrum also relates to the issue of adaptive repair and maladaptive repair after AKI. It would be interesting to identify the key factors that stop persistent activation of the above genes and determine how much EGR1 involves in those processes.

The present study has some limitations and drawbacks. First, because we mainly focused on observing changes in proliferation after AKI, the main experimental observation time was 3 d after AKI. Therefore, we did not monitor the long-term prognosis 14 d or 28 d after AKI. Second, our study did not explicitly examine the mechanism by which EGR1 is activated in AKI. Some reports 16, 17, 63 have shown that EGR1 expression can be induced by growth factors, hypoxia, and pro-inflammatory cytokines, but it remains unclear why EGR1 expression increased so quickly and to such a great extent after AKI. These issues will be illustrated in our future research.

In summary, our findings reveal that EGR1 is induced rapidly and transiently in the renal tubular epithelium after IRI and FA-induced AKI. We demonstrated that EGR1 has a renoprotective effect through increasing SOX9 expression in renal tubular epithelial cells by binding directly to the promoter of the *Sox9* gene, further promoting SOX9 positive tubular regeneration by activating the Wnt/β-catenin pathway. This study provides novel insights into the role and mechanism of EGR1 in protecting renal tubules after AKI.

## Supplementary Material

Supplementary figures and tables.

## Abbreviations

AKI: acute kidney injury
AQP2: aquaporin 2
ATN: acute tubular necrosis
CCK-8: cell counting kit-8
CFP: cyan fluorescence protein
ChIP: chromatin immunoprecipitation
CKD: chronic kidney disease
CMV: cytomegalovirus
DAPI: 4' ,6-diamidino-2-phenylindole
DBA: dolichos biflorus agglutinin
DEGs: differentially expressed genes
DKD: diabetic kidney disease
EdU: ethynyl deoxyuridine
EGR1: early growth response 1
*Egr1^Pax8-OV^*: *Egr1* overexpression plasmid with Pax8 promoter
*Egr1^CMV-OV^*: *Egr1* overexpression plasmid with CMV promoter
*Egr1^CMV-OV-CFP^*: *Egr1* overexpression plasmid with CMV promoter and cyan fluorescence protein
*Egr1^Pax8-OV-CFP^*: *Egr1* overexpression plasmid with Pax8 promoter and cyan fluorescence protein
EMSA: electrophoretic mobility shift assay
ESRD: end-stage renal disease
FA: folic acid
FC: fold change
FDR: false discovery rate
GEO: gene expression omnibus
GSEA: gene set enrichment analysis
H/R: hypoxia/reoxygenation
IRI: ischemia/reperfusion injury
KIM1: kidney injury molecule-1, a marker of kidney injury
LTL: lotus teragonolobus lectin
mIF: multiplex immunofluorescence
OCT: optimal cutting temperature compound
PAS: periodic acid-schiff
*Pax8*: paired box 8 gene
PBS: phosphate buffered saline
PCNA: proliferating cell nuclear antigen
PFA: paraformaldehyde
PMSF: phenylmethanesulfonyl fluoride
PNA: peanut agglutinin
pRL-CMV: renilla luciferase's reporter plasmid
qRT-PCR: quantitative real-time PCR
RFL: relative firefly luciferase
RIPA: radio immunoprecipitation assay
RNA-Seq: RNA sequencing
SCr: serum creatinine
*siEgr1*: silencing *Egr1* expression
*siRNA*: small interfering RNA
SOX9: sex-determining region Y box 9
STRING: the search tool for the retrieval of interacting genes/proteins
TCMK1: a mouse kidney epithelial cell line
TEC: tubular epithelial cell
TSA: tyramine signal amplification
