# Supplementary material for: Transient upregulation of EGR1 signaling enhances kidney repair by activating SOX9+ renal tubular cells
Source: Theranostics. 2022 Jul 11;12(12):5434–50. doi: 10.7150/thno.73426 (PMC9330523; doi:10.7150/thno.73426)
Supplement: Supplementary file 1 — Supplementary figures and tables. [file thnov12p5434s1.pdf]

SUPPLEMENTAL INFORMATION

Supplemental table

Table S1. Gene-specific primers used in our study.

Genes	Forward Primer（5' to 3'）	Reverse Primer（5' to 3'）	Product Length
18s	GTAACCCGTTGAACCCCATT (NR_003278.3,1577-1596)	CCATCCAACGGTAGTAGCG (NR_003278.3,1726-1708)	150bp
Egr1	CCACCTTACCACCCACATCC (NM_007913.5, 1419-1438)	AGGCCACCACACTTTTGTCT (NM_007913.5, 1573-1554)	155bp
Sox9	GTGCAAGCTGGCAAAGTTGA (NM_011448.4,1108-1127)	TGCTCAGTTCACCGATGTCC (NM_011448.4,1213-1194)	106bp
CyclinD1	AGAGGCGGATGAGAACAAGC (NM_007631.3, 645-664)	CAGTCCGGGTCACACTTGA (NM_007631.3, 866-848)	222bp
cMyc	CAGTGGTCTTTCCCTACCCG (NM_001177353.1, 1201-1220)	GGAGAGAAGGCCGTGGAATC (NM_001177353.1, 1279-1260)	79bp
Kim1	ACATATCGTGGAATCACAACGAC (NM_134248.2, 130-152)	ACAAGCAGAAGATGGGCATTG (NM_134248.2, 189-169)	60bp

Table S2. Antibodies used for Western blot analysis and immunofluorescence staining.

**Western Blot Analysis**	The primary antibodies used were as follows: anti-EGR1 (MA5-15008, Invitrogen, Thermofisher, USA), anti-SOX9 (ab185966, Abcam) and anti-GAPDH (AF0006, Beyotime Biotechnology, China). The second antibodies were HRP-labeled goat anti-rabbit IgG (H + L) (A0208, Beyotime Biotechnology, China) and HRP-labeled Goat Anti-Mouse IgG(H+L) (A0216, Beyotime Biotechnology, China).
**Immunofluorescence staining and Multiplex immunofluorescence staining**	The primary antibodies used were as follows: anti-EGR1 (MA5-15008, Invitrogen, Thermofisher, USA), anti-SOX9 (ab185966, Abcam), anti-KIM-1(AF1817, R&D systems), anti-PCNA (ab29, abcam), anti-PAX2 (21385, protentech), anti-KI67 (ab15580, abcam), anti-EMCN (Endomucin, ab106100, abcam), anti-AQP2(sc-515770, Santa cruz, a marker of the collecting duct). Three fluorescein labeled antibodies, LTL (fluorescein labeled Lotus Tetragonolobus Lectin, a marker of the proximal tubule, FL-1321, Vector Labs), DBA (fluorescein labeled Dolichos biflorus agglutinin, a marker of the collecting duct, FL-1031, Vector Labs), PNA (fluorescein labeled peanut agglutinin, marker of the loop of Henle/distal tubule) (FL-1071, Vector Labs).

Table S3. Gene-specific siRNA oligo sequence used in our study.

Genes	Sense	Antisense
siCon	5’-UUCUCCGAACGUGUCACGUTT-3’	5’-ACGUGACACGUUCGGAGAATT-3’
si*Egr1*	5’-GGACAAGAAAGCAGACAAATT-3’	5’-UUUGUCUGCUUUCUUGUCCTT-3’
si*Sox9*	5’-GGAACAACCAGUCUACACATT -3’	5’-UGUGUAGACUGGUUGUUCCTT -3’

Table S4. Predicted binding sites between the transcription factor EGR1 and the *Sox9* promoter region.

**Name**	**Score**	**Relative score**	**Start**	**End**	**Strand**	**Predicted sequence**
EGR1	9.30485	0.841192372	2154	2164	-	AGCGGAGGAGG
EGR1	9.19768	0.838494473	1568	1578	+	AGTGGGGGTGG
EGR1	8.95362	0.832350306	840	850	-	CAGGTGGGCGT
EGR1	7.96146	0.807373107	1624	1634	-	TGTGTGTGTGT
EGR1	7.96146	0.807373107	1626	1636	-	TGTGTGTGTGT
EGR1	7.96146	0.807373107	1628	1638	-	TGTGTGTGTGT
EGR1	7.96146	0.807373107	1630	1640	-	TGTGTGTGTGT
EGR1	7.96146	0.807373107	1632	1642	-	TGTGTGTGTGT
EGR1	7.96146	0.807373107	1634	1644	-	TGTGTGTGTGT
EGR1	7.96146	0.807373107	1636	1646	-	TGTGTGTGTGT
EGR1	7.96146	0.807373107	1648	1658	-	TGTGTGTGTGT
EGR1	7.96146	0.807373107	1650	1660	-	TGTGTGTGTGT
EGR1	7.96146	0.807373107	1652	1662	-	TGTGTGTGTGT
EGR1	7.96146	0.807373107	1654	1664	-	TGTGTGTGTGT
EGR1	7.96146	0.807373107	1656	1666	-	TGTGTGTGTGT
EGR1	7.96146	0.807373107	1658	1668	-	TGTGTGTGTGT
EGR1	7.96146	0.807373107	1660	1670	-	TGTGTGTGTGT
EGR1	7.96146	0.807373107	1662	1672	-	TGTGTGTGTGT
EGR1	7.96146	0.807373107	1664	1674	-	TGTGTGTGTGT
EGR1	7.96146	0.807373107	1666	1676	-	TGTGTGTGTGT
EGR1	7.96146	0.807373107	1668	1678	-	TGTGTGTGTGT
EGR1	7.96146	0.807373107	1670	1680	-	TGTGTGTGTGT
EGR1	7.96146	0.807373107	1672	1682	-	TGTGTGTGTGT
EGR1	7.96146	0.807373107	1674	1684	-	TGTGTGTGTGT
EGR1	7.93693	0.806755478	1303	1313	+	TGCGGGAGCGC
Sox9 promoter region sequence (Sequence ID: NC_000077.6:112780210-112782209), transcription factor EGR1 (matrix ID: MA0162.1)						

Supplemental Figures & Legends

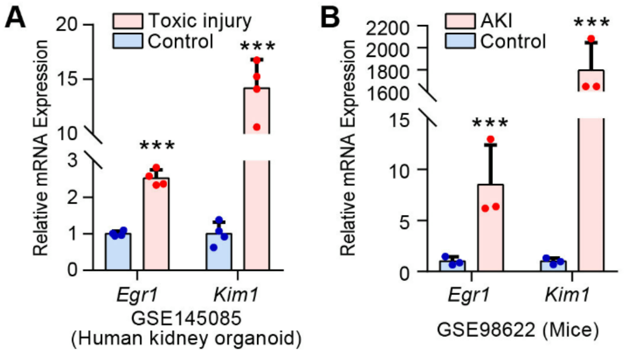

Figure S1. *Egr1* and *Kim1* mRNA expression were significantly elevated in human kidney organoid with Toxic injury and mice AKI. (A) *Egr1* and *Kim1* mRNA expression in GSE145085 datasets. **(B)** Expression of *Egr1* and *Kim1* mRNA in GSE53769 datasets. ***p < 0.001. AKI, acute kidney injury.

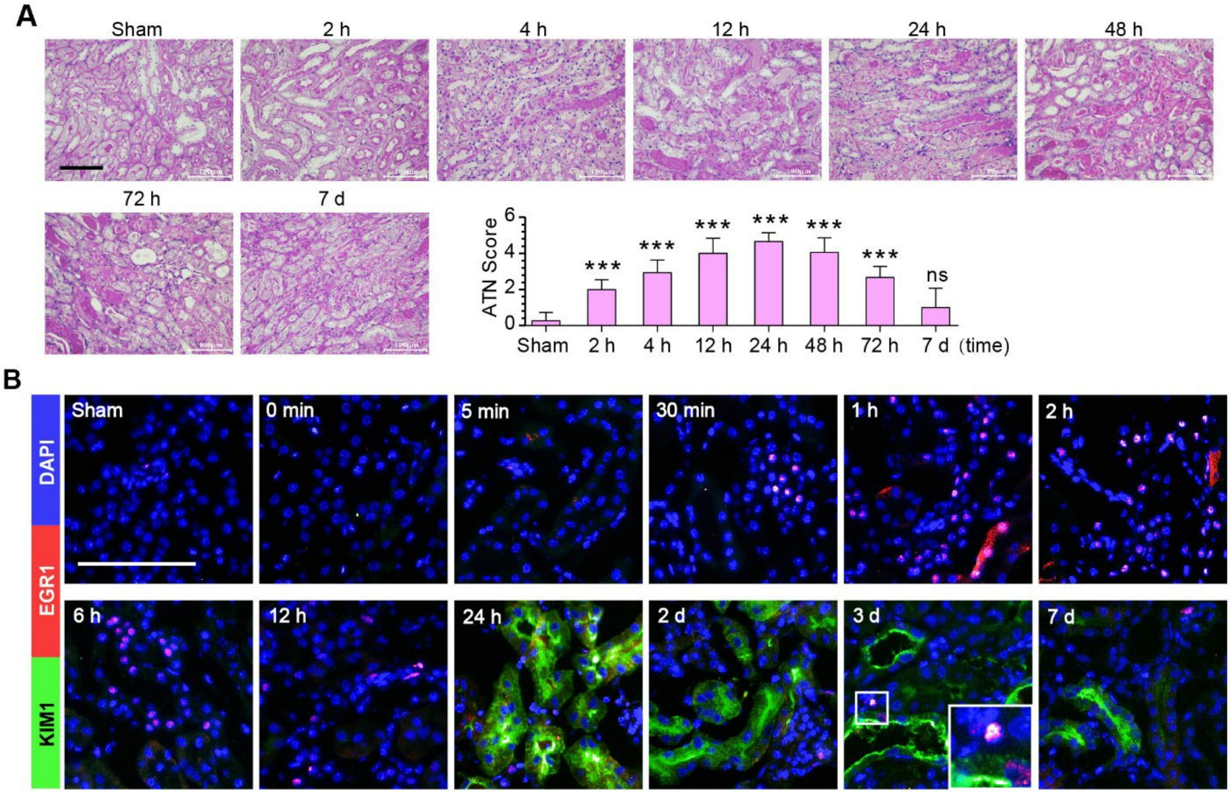

Figure S2. EGR1 is transiently and rapidly induced in ischemic AKI. (A) PAS staining of ischemic AKI induced by IRI, and the evaluation of the severity of renal injury (ATN score) from PAS staining. **(B)** Double immunofluorescence staining of KIM1 and EGR1 expression in renal tissues at different reperfusion time points (including 5min and other super-early stages) after 30min ischemia. The image of 3d group was enlarged to see whether KIM1 and EGR1 can be co-staining, it can be seen that EGR1 is less expressed on IRI 3d and also few co-staining with KIM1. Scale bars: 100 µm. ns indicates $p < 0.05$, ***$p < 0.001$. AKI, acute kidney injury; IRI, ischemia-reperfusion injury; PAS, periodic acid-schiff; ATN, acute tubular necrosis.

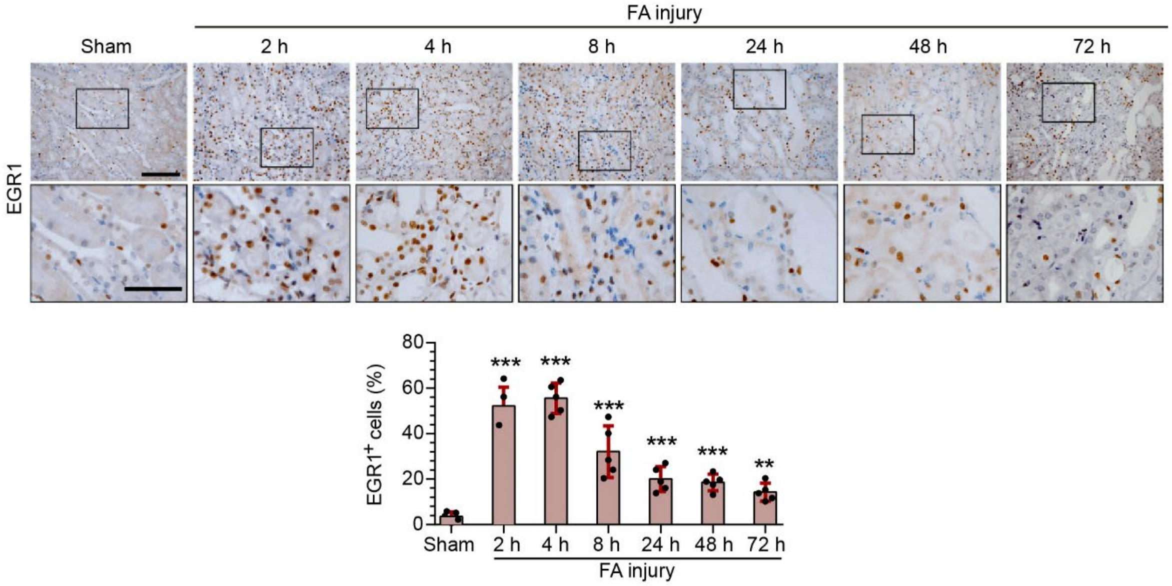

Figure S3. EGR1 is induced in toxic AKI. Representative micrographs show the expression of EGR1 expression at different time points of kidney FA injury determined by immunohistochemistry. n = 5 mice per group. Scale bars: 100 μm (upper panel), 50 μm (lower panel). $^{**}p < 0.01$, $^{***}p < 0.001$. FA, folic acid; AKI, acute kidney injury.

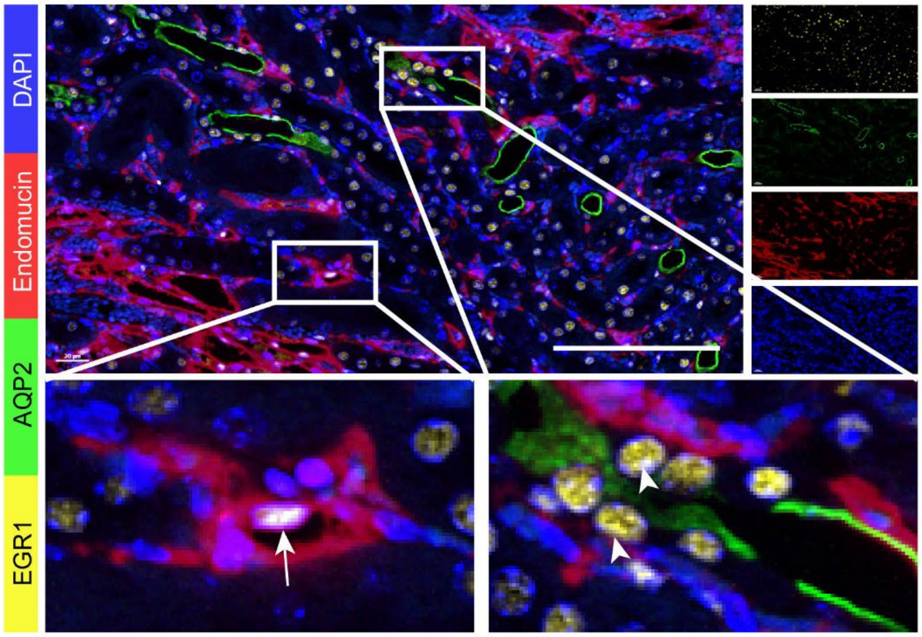

Figure S4. Multiplex immunofluorescence (mIF) staining of EGR1 with AQP2, and Endomucin after IRI. Kidney cryosections 2 h after IRI were sequentially stained with EGR1, AQP2, Endomucin, and DAPI. The arrow indicates co-staining of EGR1 with Endomucin, and the sessile arrow indicates co-staining of EGR1 with AQP2. Scale bars: 100 μm. mIF, multiplex immunofluorescence; IRI, ischemia-reperfusion injury; AQP2, aquaporin 2, a collecting duct marker; Endomucin, an endothelial cell marker; DAPI, 4’ ,6-diamidino-2-phenylindole.

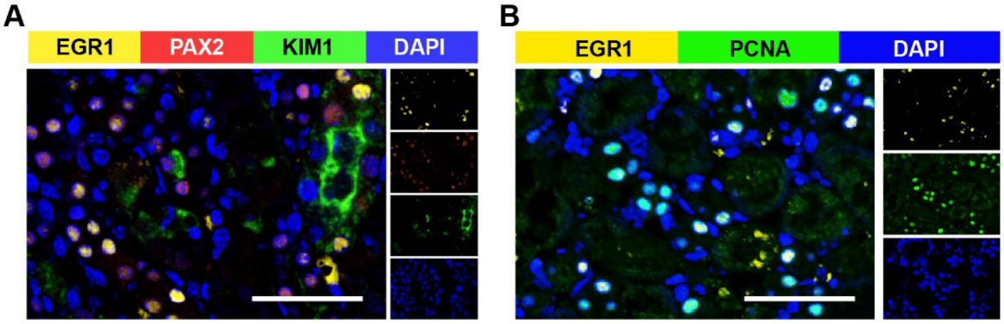

Figure S5. Multiplex immunofluorescence (mIF) staining of EGR1 with PAX2, KIM1, or PCNA after IRI. (A) Kidney cryosections 3d after IRI (mice injected with $\mathit{Egr1}^{\mathit{Pax8\text{-}OV}}$ plasmid) and were sequentially mIF stained with EGR1, PAX2, KIM1, and DAPI. **(B)** Kidney cryosections 3d after IRI (mice injected with $\mathit{Egr1}^{\mathit{Pax8\text{-}OV}}$ plasmid) and were sequentially mIF stained with EGR1, PCNA, and DAPI. Scale bars: 50 µm. mIF, multiplex immunofluorescence; IRI, ischemia-reperfusion injury; KIM1, a marker of kidney injury; PAX2, a marker of dedifferentiated proximal tubule cells; PCNA, a marker of cell proliferation; DAPI, 4' ,6-diamidino-2-phenylindole. $\mathit{Egr1}^{\mathit{Pax8\text{-}OV}}$, *Egr1* overexpression plasmid with *Pax8* promoter.

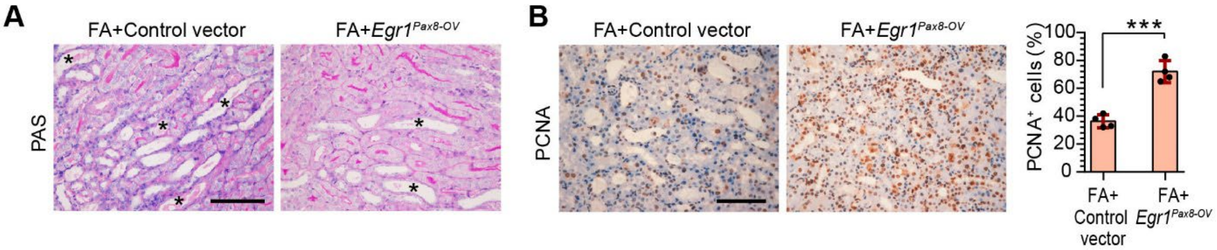

Figure S6. EGR1 decreases tubular injury and drives renal tubule repair and regeneration in FA-AKI. (A) Representative micrographs after PAS staining show kidney injury in mice injected with Vehicle or *pPax8-Egr1* plasmid ($Egr1^{Pax8\text{-}OV}$) 3 days after FA injury. The asterisks in the enlarged boxed areas indicate injured tubules. **(B)** Representative micrographs and quantitative data showing the number of PCNA-positive tubular cells in different groups after FA injury. n = 4 mice per group. Scale bars: 100 µm. ****p* < 0.001. AKI, acute kidney injury; FA, folic acid; $Egr1^{Pax8\text{-}OV}$, *Egr1* overexpression plasmid with *Pax8* promoter.

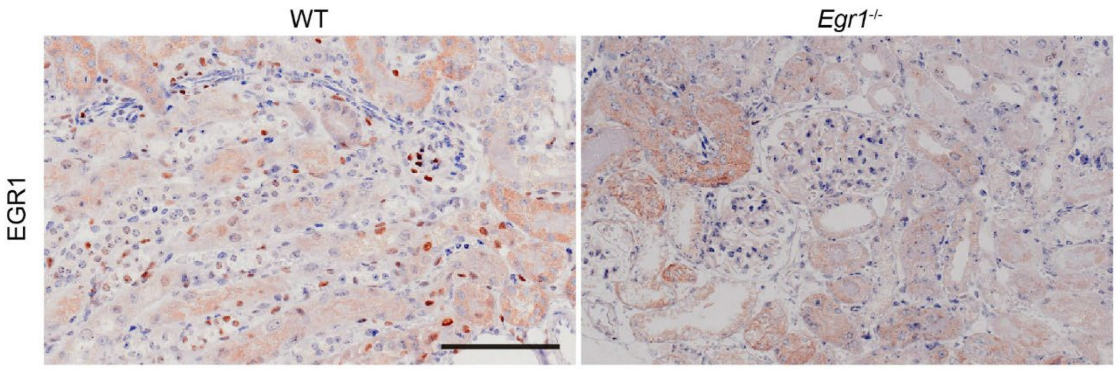

Figure S7. Micrographs showing effective knockout of the EGR1 protein in kidneys with IRI. The efficacy of *Egr1* knockout was confirmed by immunohistochemistry 2 h after IRI, and *Egr1* was barely detected in *Egr1*$^{-/-}$ mice. Scale bars: 100 μm. WT mice, wild-type mice; *Egr1*$^{-/-}$ mice, *Egr1* knockout mice; IRI, ischemia-reperfusion injury.

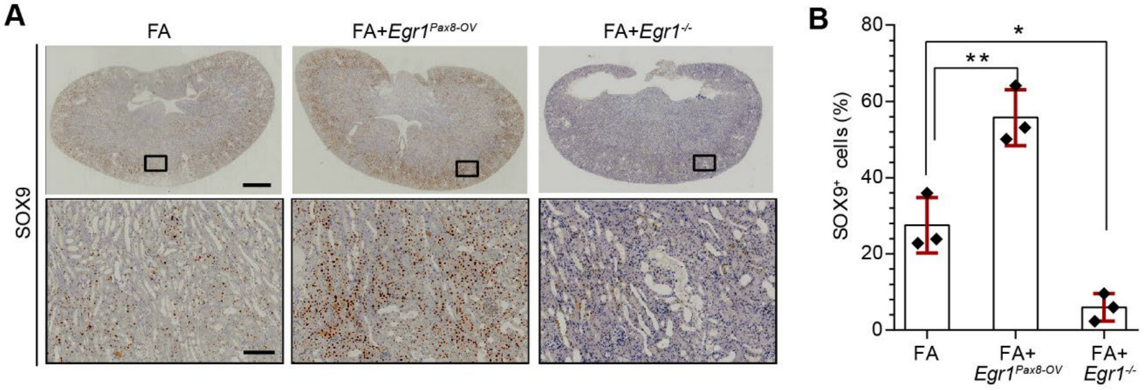

Figure S8. EGR1 promotes SOX9 expression after FA-AKI. (A) Immunohistochemical analysis and **(B)** quantitative SOX9 expression data in different groups after FA injury. *$p < 0.05$; **$p < 0.01$, ANOVA corrected for Bonferroni coefficient. n = 3 per group. Scale bars: 1mm (upper part), 100 µm (lower part). AKI, acute kidney injury; *Egr1*$^{Pax8\text{-}OV}$, *Egr1* overexpression plasmid with *Pax8* promoter; FA, folic acid; *Egr1*$^{-/-}$ mice, *Egr1* knockout mice.

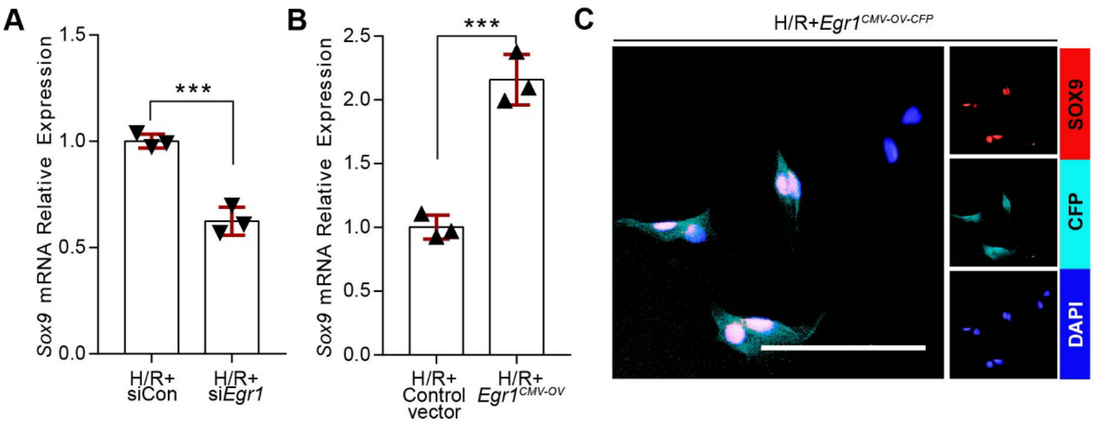

Figure S9. EGR1 promotes SOX9 expression *in vitro*. (A) The expression level of *Sox9* mRNA decreased after *Egr1* knockdown in TCMK1 cells subjected to H/R **(B)** but increased after Egr1 overexpression in TCMK1 cells subjected to H/R. **(C)** Representative immunofluorescence image showed that EGR1 was co-stained with SOX9 after $Egr1^{CMV\text{-}OV\text{-}CFP}$ plasmid transfected in TCMK1 cells which subjected to H/R. Scale bars: 100 μm. ***$p < 0.001$, unpaired Student's t test without pairing. n = 3 per group. H/R, hypoxia/reoxygenation; siCon, negtive control small interfering RNA; CFP, cyan fluorescence protein; $Egr1^{CMV\text{-}OV\text{-}CFP}$, *Egr1* overexpression plasmid with CMV promoter and cyan fluorescence protein.

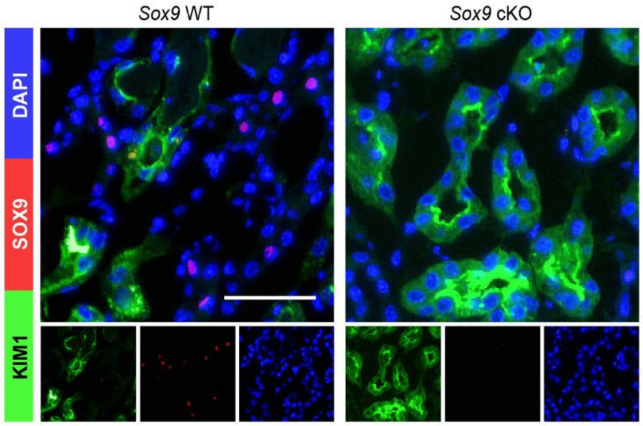

Figure S10. Micrographs show the effective knockout of the SOX9 protein in kidneys with IRI. The efficacy of *Sox9* knockout was confirmed by immunohistochemistry 3d after IRI, and *Sox9* was barely detected in *Sox9cKO* mice. Scale bars: 50 μm. IRI, ischemia-reperfusion injury; *Sox9* WT mice, $Slc34a1^{CreERT2/+}$:$Sox9^{+/+}$ mice; *Sox9* cKO mice, $Slc34a1^{CreERT2/+}$:$Sox9^{fl/fl}$ mice.

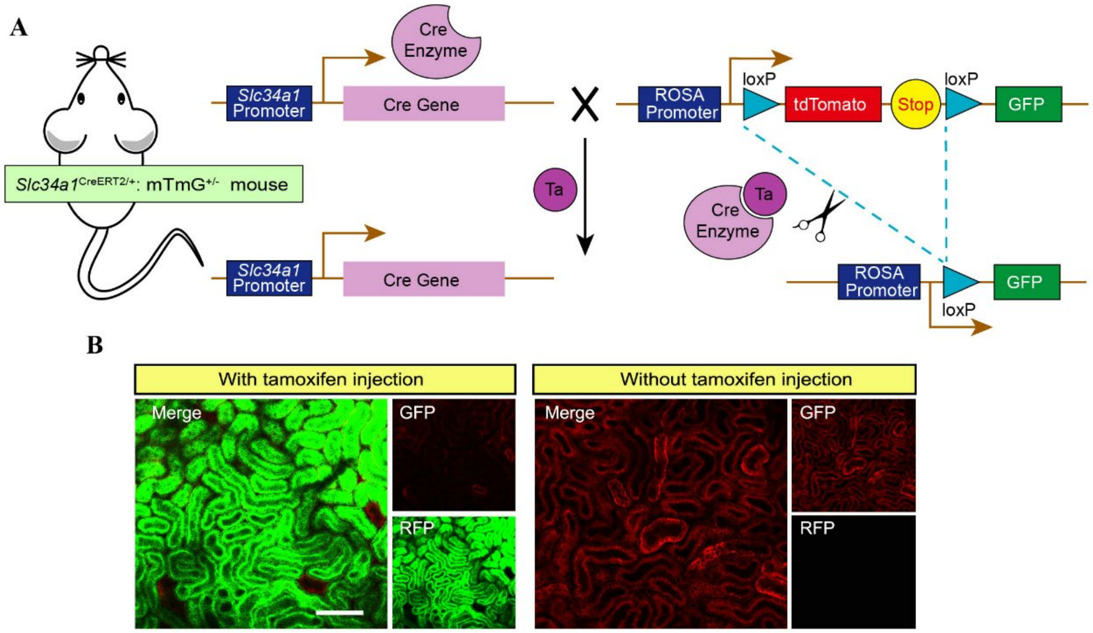

Figure S11. The efficiency of the Cre recombinant enzyme. **(A)** Breeding scheme. **(B)** After giving Slc34a1$^{CreERT2/+}$; mTmG$^{+/-}$ mice a peritoneal injection of tamoxifen (120 mg/kg, once per 2 d, 3 times) to activate the catalytic activity of the inducible Cre enzyme, the activity of the recombinant enzyme reached almost 100%, that is, the RFP cells almost 100% transformed into GFP cells. Scale bars: 100 μm. Ta, Tamoxifen; RFP, Red fluorescent protein; GFP, Green fluorescent protein.
